# Oleoylethanolamide attenuates cocaine-primed reinstatement and alters dopaminergic gene expression in the striatum

**DOI:** 10.1186/s12993-023-00210-1

**Published:** 2023-05-24

**Authors:** Macarena González-Portilla, Susana Mellado, Sandra Montagud-Romero, Fernando Rodríguez de Fonseca, María Pascual, Marta Rodríguez-Arias

**Affiliations:** 1 Department of Psychobiology, Facultad de Psicología, Universitat de València, Avda. Blasco Ibáñez 21, 46010 Valencia, Spain; 2 Department of Physiology, School of Medicine, Universitat de Valencia, Avda. Blasco Ibáñez, 15, 46010 Valencia, Spain; 3 Mental Health Clinical Management Unit, Institute of Biomedical Research of Malaga- IBIMA, Regional University Hospital of Málaga, 29010 Málaga, Spain; 4 Atención primaria, cronicidad y promoción de la salud, Red de investigación en atención primaria de adicciones (RIAPAD), Rd210009/0005/0003 Valencia, Madrid, Spain

**Keywords:** Oleoylethanolamide, Cocaine, Reinstatement, Gene expression, Dopamine

## Abstract

The lipid oleoylethanolamide (OEA) has been shown to affect reward-related behavior. However, there is limited experimental evidence about the specific neurotransmission systems OEA may be affecting to exert this modulatory effect. The aim of this study was to evaluate the effects of OEA on the rewarding properties of cocaine and relapse-related gene expression in the striatum and hippocampus. For this purpose, we evaluated male OF1 mice on a cocaine-induced CPP procedure (10 mg/kg) and after the corresponding extinction sessions, we tested drug-induced reinstatement. The effects of OEA (10 mg/kg, i.p.) were evaluated at three different timepoints: (1) Before each cocaine conditioning session (OEA-C), (2) Before extinction sessions (OEA-EXT) and (3) Before the reinstatement test (OEA-REINST). Furthermore, gene expression changes in dopamine receptor D1 gene, dopamine receptor D2 gene, opioid receptor µ, cannabinoid receptor 1, in the striatum and hippocampus were analyzed by qRT-PCR. The results obtained in the study showed that OEA administration did not affect cocaine CPP acquisition. However, mice receiving different OEA treatment schedules (OEA-C, OEA-EXT and OEA-REINST) failed to display drug-induced reinstatement. Interestingly, the administration of OEA blocked the increase of dopamine receptor gene D1 in the striatum and hippocampus caused by cocaine exposure. In addition, OEA-treated mice exhibited reduced striatal dopamine receptor gene D2 and cannabinoid receptor 1. Together, these findings suggest that OEA may be a promising pharmacological agent in the treatment of cocaine use disorder.

## Introduction

Among psychostimulants, cocaine is the most abused substance worldwide with an ongoing increasing tendency over the past decade [1]. Epidemiological studies estimate that there are approximately 1.3 million people diagnosed with cocaine use disorder (CUD) in the US. Although there has been promising progress in the development of effective treatments, to date, no pharmacological therapy has been approved for CUD [2].

One of the major hallmarks of substance use disorder, including CUD, is a high risk of relapse following treatment, even after long periods of abstinence [3]. Vulnerability to relapse is markedly increased when a recovering individual experiences high levels of stress, encounters contextual cues associated with prior drug use oringests a low dose of the drug [4]. In the laboratory, the mechanisms that underlie the persistent risk of relapse can be studied using rodent models of reinstatement. In the conditioned place preference paradigm (CPP), the reinstatement test is performed after the extinction of the drug-reinforced place preference. One of the strategies used to reinstate drug seeking is drug-priming with a small dose of the drug. This approach has been shown to be very useful in finding potential pharmacological agents that reduce cocaine relapse risk [5, 6].

Research has identified multiple neuronal mechanisms that contribute to the lasting vulnerability to relapse into cocaine use. The main effect of cocaine intake is an acute inhibition of monoamine reuptake in the nucleus accumbens, a key brain region of the mesolimbic pathway [7]. It is now established that repeated cocaine exposure induces long-term neuronal adaptations in the mesolimbic system, which contribute to persistent drug seeking, craving and relapse [8]. Indeed, this dopaminergic signaling is necessary for a stressful stimulus, contextual cues, or cocaine priming to induce reinstatement behavior [9]. Mounting evidence has shown that other non-dopaminergic systems are involved in relapse vulnerability. Recently, the endocannabinoid system has been revealed as an important modulator of dopaminergic signaling and cocaine reward [10–13]. Studies with rodents have shown that administration of cannabinoid antagonists results in diminished cocaine reward and attenuated cocaine-primed and cue-induced reinstatement of cocaine-seeking behavior [14, 15]. In parallel, numerous studies have highlighted the involvement of the opioid system in drug relapse. More specifically, several lines of research indicate a crucial role for the µ opioid receptor in the neurocircuitry that mediates stress-induced reinstatement of cocaine seeking behavior [16, 17].

Research has shown that active compounds that target the aforementioned brain systems can alleviate the negative outcomes of cocaine use and thus, reduce relapse susceptibility. In the past years, there has been increasing evidence showing that lipid-based signaling molecules are important regulators of reward and drug-seeking behavior [18]. Oleoylethanolamide (OEA) is a lipid belonging to the N-acetylethanolamine family (NAEs) present in most mammals [19]. In humans, OEA is synthesized by cells in the small intestine and adipose tissue. The vagal afferent fibers allow for communication from the intestine to the CNS [20]. In the brain, OEA has neuromodulatory effects by binding to nuclear receptor peroxisome proliferator-activated receptor alpha (PPAR-α) and the capsaicin receptor transient receptor potential cation channel subfamily V member 1 (TRPV1) [21, 22]. Anatomical studies have shown that PPAR-α and TRPV1 are widely expressed in the mesolimbic system, including the striatum and hippocampus, which are known to be brain areas involved in the reinstatement of drug-seeking behavior [23–26].

Recently, preclinical studies have found that OEA also modulates reward-related behavior including cocaine-induced behaviors [18, 27, 28, 30]. Bilbao and co-workers [31] showed that OEA administration reduced psychomotor activation induced by cocaine and blocked cocaine CPP. Similarly, a recent study has found that OEA treatment blocks stress-induced cocaine CPP [32]. Furthermore, cocaine self-administration has been shown to affect OEA levels in limbic areas such as the dorsal striatum [33, 34].

The available evidence on the neuronal pathways underlying the modulatory effect of OEA on cocaine reward is limited and scattered. To exert these various effects on cocaine-related behavior OEA may be targeting multiple brain signaling pathways. Thus, the aim of this study was to evaluate the effects of OEA on cocaine reward by using the CPP paradigm. In addition, gene expression analyses were carried out by quantitative real-time polymerase chain reaction (qRT-PCR) to evaluate changes in relevant signaling targets involved in cocaine relapse, including dopamine receptor D1 gene (DrD1), dopamine receptor D2 gene (DrD2), opioid receptor µ (OPRM1), and cannabinoid receptor 1 (CNR1). We analyzed brain structures affected in the pathophysiology of drug abuse, i.e., the striatum, which regulates reward processing, and the hippocampus, which plays a key role in memory formation and learning.

## Methods and materials

### Animals and experimental design

OF1-strain adult male mice (n = 56) were used in this study (Charles River, France). On arrival, mice were housed in groups of four in plastic cages under constant temperature under a reverse 12-h light/dark cycle and water and food available *ad libitum*, except during behavioral testing. All animals acclimated to the environment for one week before the experimental procedure.

**Fig. 1:**
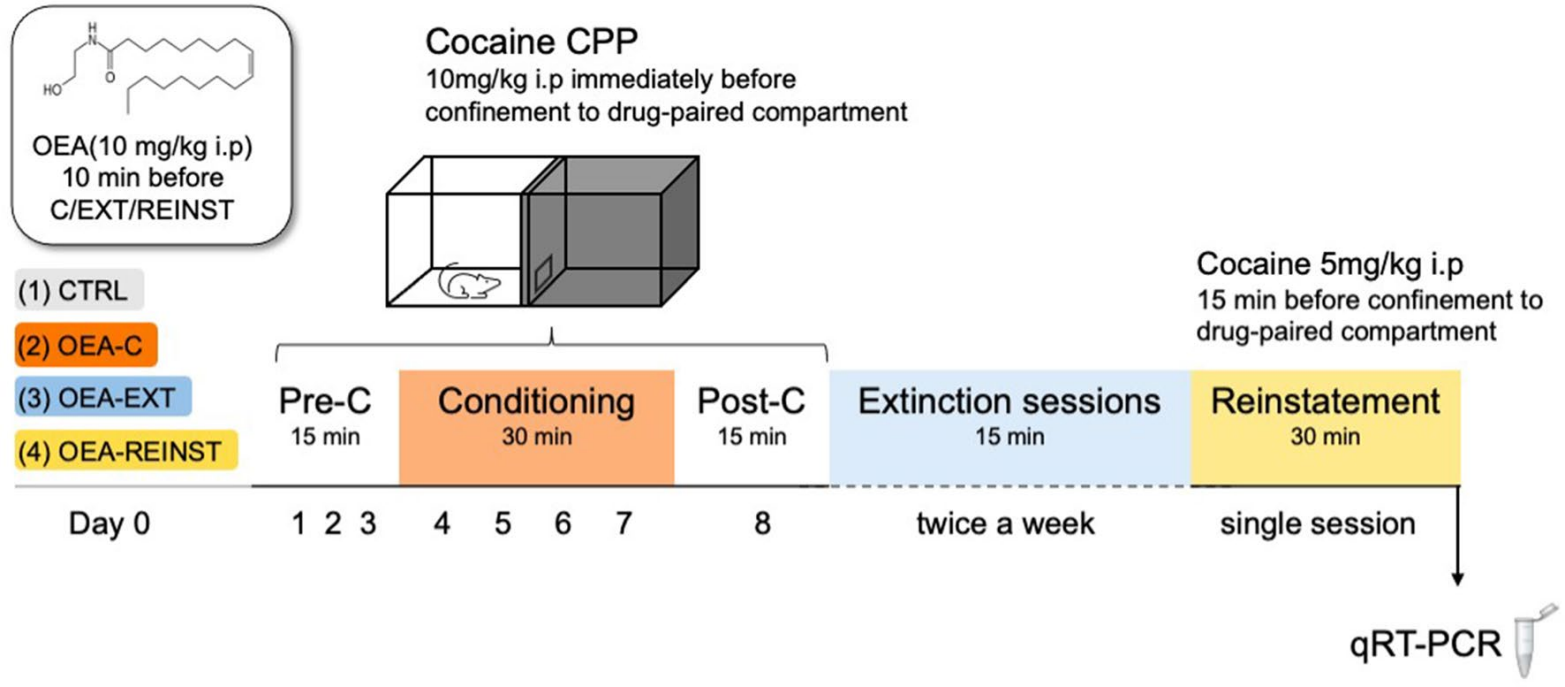
**A** Experimental design. Mice were divided into different experimental groups according to OEA treatment schedules: (1) CTRL; control (2) OEA-C; received OEA before each cocaine conditioning session, (3) OEA-EXT received OEA before each extinction session and (4) OEA-REINST received OEA before the reinstatement test. OEA administration consisted of OEA i.p (10 mg/kg) 10 min before each of the corresponding timepoints specified above

Mice were divided into different experimental groups according to different OEA treatment schedules: (1) Control CTRL (2) received OEA i.p (10 mg/kg) before each cocaine conditioning session (OEA-C), (3) before each extinction sessions (OEA-EXT) and (4) before the reinstatement test (OEA-REINST), (see Fig. 1). All procedures were conducted in compliance with the guidelines of the European Council Directive 2010/63/EU regulating animal research and were approved by the local ethics committees of the University of Valencia.

### Drug administration

OEA (10 mg/kg, i.p.; synthesized as described in [34]) was dissolved in 5% Tween 80 in saline and injected 10 min before the corresponding test. The doses were chosen according to previous studies in rodents reporting effective therapeutic effects [35–38].

For CPP, animals were injected with 10 mg/kg cocaine hydrochloride (Laboratorios Alcaliber S.A., Madrid, Spain) diluted in saline (NaCl 0.9%) and adjusted to a volume of 0.01 ml/g of weight.

### Conditioning place preference (CPP)

#### Apparatus

For place conditioning, we employed Plexiglas boxes with equally sized compartments (30.7 cm length x 31.5 cm width x 34.5 cm height) separated by a gray central area. The compartments had different colored walls (black or white) and distinct floor textures (fine grid in the black compartment and wide grip in the white one). Four infrared light beams in each compartment of the box and six in the central area allowed the recording of the position of the animal and the number of crossings from one compartment to the other. The equipment was controlled by two IBM PC computers using MONPRE 2Z software (CIBERTEC S.A., Spain.).

#### 10 mg/kg cocaine-induced CPP

A three-stage CPP procedure consisting of acquisition, extinction and reinstatement was performed. The CPP acquisition was performed as described previously [12] and consisted of three phases (see Fig. 1). During the first phase (Pre-Conditioning; Pre-C), mice were allowed access to both compartments of the apparatus for 15 min (900 s) per day for 3 days. On day 3, the time spent in each compartment over a 900-s period was recorded, and animals showing a strong unconditioned aversion (less than 30% of the time) or preference (more than 70% of the time) for any compartment were excluded from the study (n = 15). After assigning the compartments, no significant differences were detected between the time spent in the drug-paired and vehicle-paired compartments during the pre-conditioning phase. During the second phase (Conditioning), mice received intraperitoneal injections of 1 mg/kg cocaine or saline and confined to alternating sides of the CPP apparatus. For four days, animals received an injection of saline immediately before being confined to the vehicle-paired compartment for 30 min. After an interval of 4 h, animals received an injection of cocaine immediately before being confined to the drug-paired compartment for 30 min. Confinement was carried out in both cases by blocking the access that separated the two compartments. On the Post-conditioning testing day and subsequent days, mice were allowed to move freely between sides during a 900-s recording period. For extinction, mice were placed in the CPP apparatus daily and the time spent in each compartment was measured to determine if cocaine-induced preference had disappeared. Although the mean of the group as a whole determined the day on which extinction was considered to have been achieved, preference was considered to be extinguished when a mouse spent 378 s or less in the drug-paired compartment on two consecutive days. We chose this time based on the values of all the Pre-C tests performed in the study (mean = 368 s). When the preference was not extinguished in an animal, it was assigned the number of days required for extinction for the group as a whole. Finally, 24 h after reaching the extinction criterion, mice were challenged with a cocaine injection once, followed by a place preference test (reinstatement test).

### Tissue sampling and biochemical analyses

Mice were sacrificed by cervical dislocation. The striatum and hippocampus were precisely dissected out based on the atlas of the Paxinos and Franklin [39] using a coronal brain matrix. Tissue samples were stored at −80ºC until the qRT-PCR assay was performed.

#### RNA isolation, reverse transcription, and quantitative RT-PCR

Striata and hippocampi were lysed in 1 mL of Tri-Reagent solution (Sigma-Aldrich, Madrid, Spain) and total RNA was isolated according to the manufacturer’s instructions. Then, the mRNA was reverse-transcribed by the NZY First-Strand cDNA Synthesis Kit (NZYTech, Lda. Genes and Enzymes, Lisbon, Portugal) following the manufacturer’s instructions. Amplification of the target and housekeeping (b-glucuronidase) genes was completed employing the Taqman Gene Expression Master Mix (Thermo Fisher Scientific, Madrid, Spain) in a LightCycler 480 System (Roche Diagnostics, Madrid, Spain). The assay codes of the primers used were Mm02620146 (DrD1), Mm00438545 (DrD2), Mm01188089 (Oprm), Mm01212171 (Cnr1) and Mm00446953 (b-glucuronidase). Data were analyzed using the LightCycler 480 relative quantification software and normalized to the amplification product of b-glucuronidase.

### Statistics

To test for the CPP acquisition, the time spent in the drug-paired compartment was analyzed with a two-way ANOVA with one between-subjects’ variable – Treatment, with four levels (CTRL, OEA-C, OEA-EXT, OEA-REINST) and one within subjects’ variable with two levels, pre- and post-CPP measurement (Pre-C and Post-C). Additionally, a one-way ANOVA was conducted to assess whether the conditioning score (defined as time spent in the drug-paired side minus the time spent in the saline-paired side) was different between groups. Post-hoc comparisons were performed by means of Bonferroni tests.

Extinction and reinstatement values were analyzed by a Student’s t-test and the time required for the preference to be extinguished in each animal was analyzed by means of the Kaplan–Meier test with Breslow (generalized Wilcoxon) comparisons [40].

The gene expression data were analyzed by a one-way ANOVA with one between variables, Treatment, with two levels (control, OEA-treated). Bonferroni post-hoc tests were also analyzed. In addition, correlation analysis between conditioning scores and gene expression was performed using Pearson’s correlation coefficient (r). Results are expressed as the mean ± SEM, and statistical significance was set at p < 0.05. Statistical analyses were performed using SPSS Statistics v28.

## Results

### OEA treatment does not block cocaine (10 mg/kg) CPP acquisition but prevents reinstatement

**Fig. 2:**
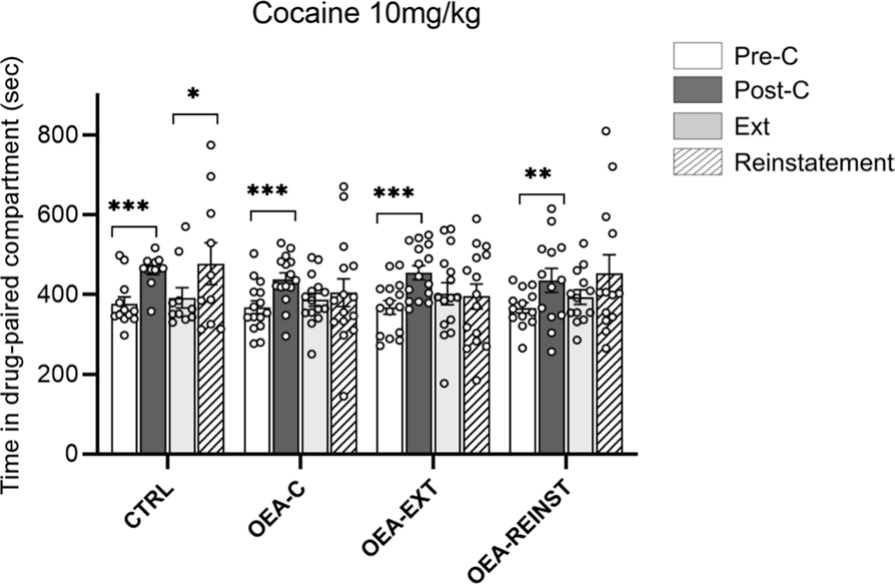
Mice were divided into the following treatment groups: Control (CTRL) (n = 12), OEA-C (n = 15) received 10 mg/kg OEA pre-treatment before each conditioning session, OEA-EXT (n = 15) received 10 mg/kg OEA pre-treatment before each extinction session and OEA-REINST (n = 13) received 10 mg/kg OEA pre-treatment before the single reinstatement test. Data presented as mean values ± S.E.M. of the time spent in the drug-paired compartment on the Pre-C test (white bars), Post-C test (dark gray bars), in the last extinction sessions (light gray bars) and during the reinstatement tests with 5 mg/kg of cocaine (striped gray bars). ***p < 0.001, ** p < 0.01, * p < 0.05 significant difference in the time spent in the drug-paired compartment vs. pre-conditioning test

The ANOVA of the CPP data revealed an effect of the variable Days (F (1,51) = 68.238; p < 0.001). Mice in every experimental group developed cocaine-induced CPP, spending more time in the drug-paired compartment during the Post-C test than in the Pre-C test (*p* < 0.001), (see Fig. 2).

**Fig. 3:**
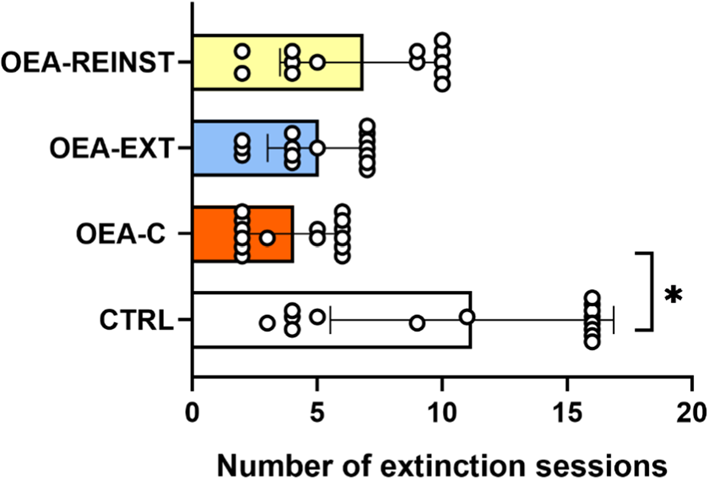
Extinction. The bars represent the total value (± S.E.M) of the number of sessions required for the preference to be extinguished after the Post-C test. * p < 0.05 significant difference with respect to CTRL group

With regards to the time required to extinguish the preference (see Fig. 3), the CTRL group required a mean number of 11.2 sessions, while the OEA-C, OEA-EXT and OEA-REINST groups required only 4, 5 and 6.8 sessions, respectively. The Kaplan-Meier analysis revealed that the CTRL group required significantly more sessions than the OEA-C to extinguish the preference (χ2 = 3.864; *p* = 0.049).

Reinstatement of drug-seeking behavior after achievement of extinction was evaluated with Student’s t-tests, which showed that reinstatement with a priming dose of 5 mg/kg cocaine was achieved only in the control group (see Fig. 2).

### OEA administration altered the relapse-related gene expression in the striatum and hippocampus

#### OEA-treated mice presented decreased striatal DrD1, DrD2, CNR1 gene expression

For DrD1 and DrD2 gene expression (see Fig. 3a, b), the ANOVA revealed a significant effect of the variable treatment [F(1,30) = 10.898; p < 0.01] and [F(1,30 = 11.007; p < 0.01], respectively. With regards to CNR1 expression, the ANOVA revealed an effect of the variable treatment [F(1,30) = 5.629; p < 0.05] (see Fig. 3d). OEA treatment induced a significant decrease in DrD1, DrD2 and CNR1 gene expression in OEA-treated mice (OEA-C, OEA-EXT and OEA-REINST groups) with respect to the CTRL group.

**Fig. 4:**
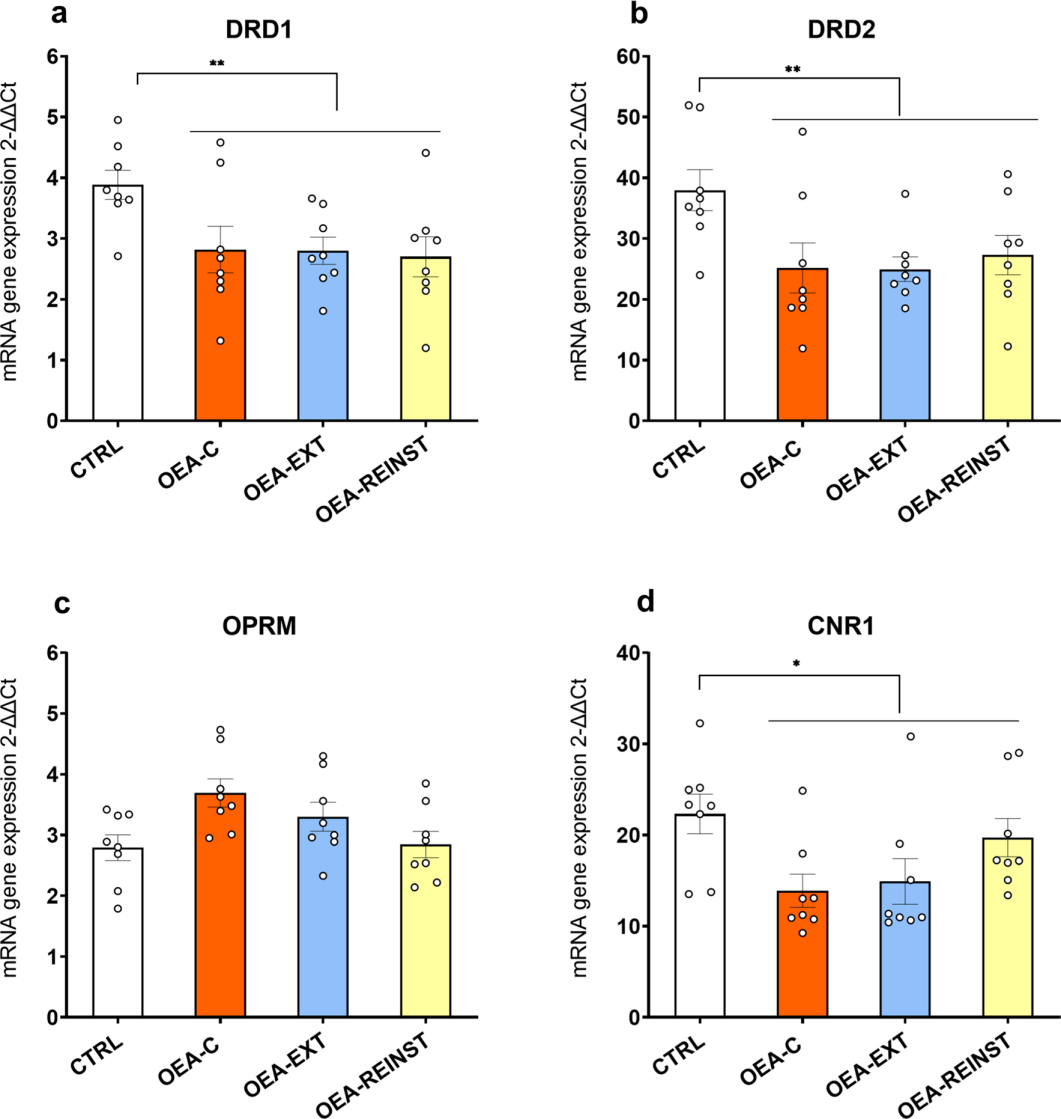
RT-PCR gene expression in the striatum (n = 8/condition). **a** Dopamine receptor D1 gene―DrD1, **b** Dopamine receptor D2 gene―DrD2, **c** Opioid receptor µ―Oprm. **d** Cannabinoid receptor 1―CNR1. The columns represent means and the vertical lines ± S.E.M of relative (2-ΔΔCt method) gene expression in the striatum of OF1 mice. * p < 0.05; ** p < 0.01 significant differences in OEA-treated mice with respect to the CTRL group

#### OEA-treated mice presented decreased hippocampal DrD1 gene expression

For DrD1 gene expression (see Fig. 4a), the ANOVA revealed a significant effect of the variable Treatment [F(1,30 = 6.525; p < 0.05]. OEA treatment significantly decreased DrD1 expression in OEA-treated mice with respect to the CTRL group.

**Fig. 5:**
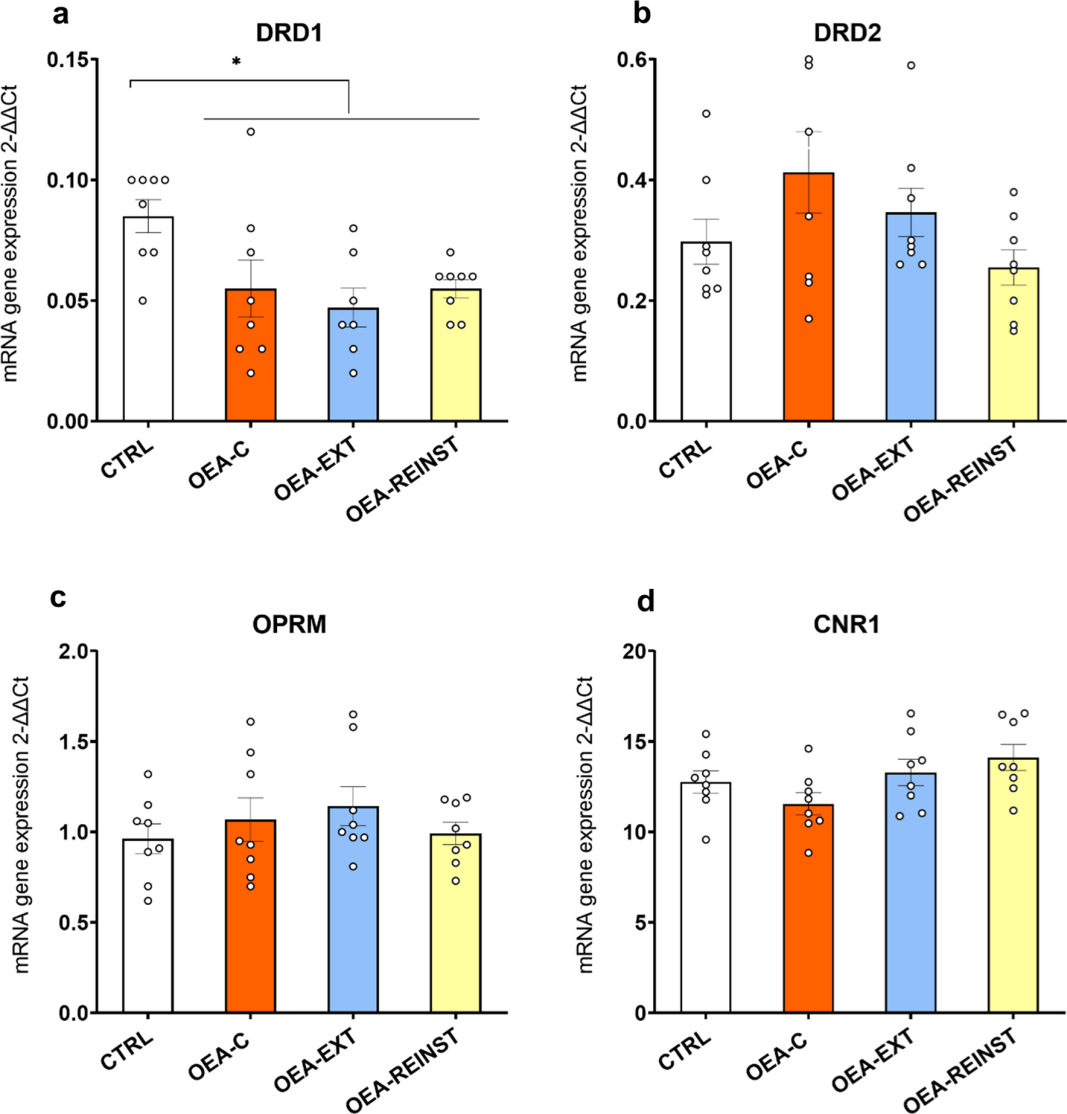
RT-PCR Gene expression in the hippocampus (n = 8/condition). **a** Dopamine receptor D1 gene―DrD1, **b** Dopamine receptor D2 gene―DrD2, **c** Opioid receptor µ―Oprm1. **d** Cannabinoid receptor 1―CNR1. The columns represent means and the vertical lines ± S.E.M of relative (2-ΔΔCt method) gene expression in the striatum of OF1 mice. * p < 0.05 significant differences with respect to the CTRL group

**Fig. 6:**
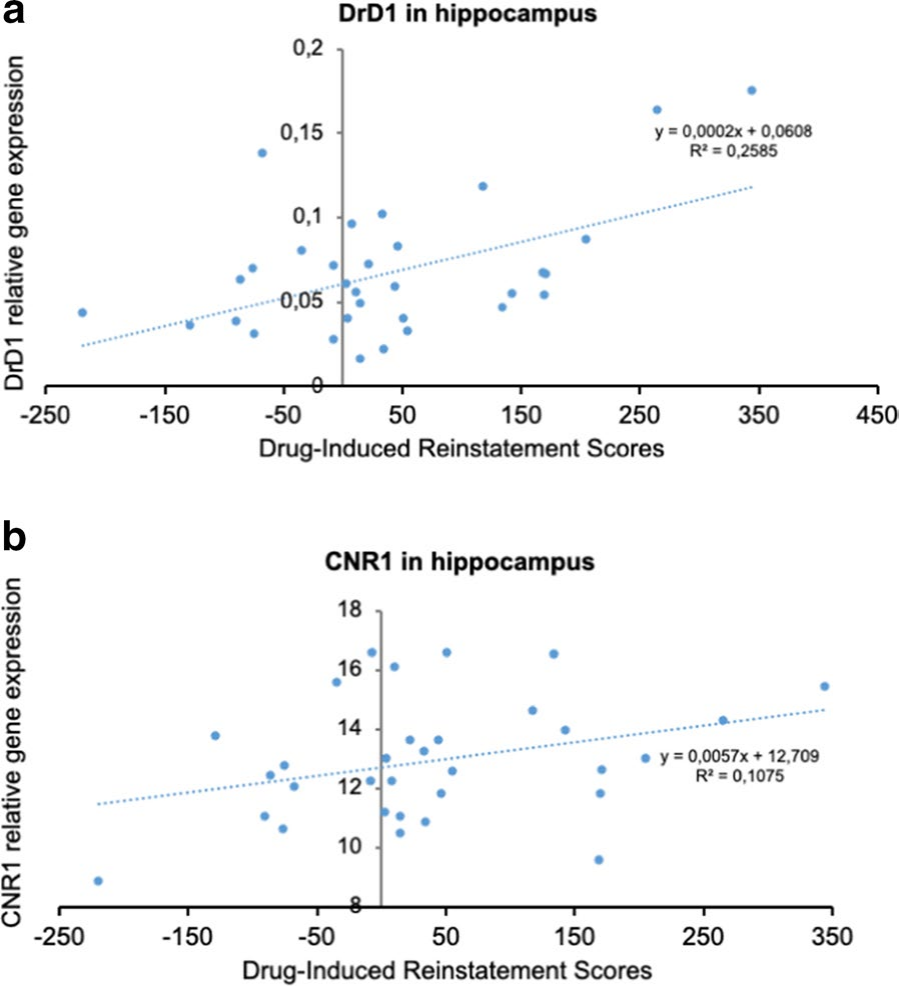
DrD1 or CNR1 gene expression within the hippocampus does not correlate with cocaine-induced CPP but it does significantly correlate positively with drug-induced reinstatement of cocaine CPP in the same mice. **A** DrD1 and **B** CNR1 in the hippocampus were measured by real-time PCR (n = 8/per experimental group)

We performed a Pearson correlation between the conditioning score after the Post-C and the reinstatement test and the expression of DrD1, DrD2, OPRM1, and CNR1 in the hippocampus and striatum (See Fig. 5). Although the conditioning score after Post-C test did not show any correlation, we obtained a positive significant Pearson correlation coefficient between the conditioning score of the reinstatement test and the hippocampal expression of DrD1 (r = 0.508, p < 0.003) and a tendency with the expression of CNR1 (r = 0.328, p < 0.067), meaning that a higher place preference in the reinstatement test positively correlates with a higher expression of these gene receptors (see Fig. 6).

Additionally, we also obtained other interesting correlations among the expression of these genes. Expression of DrD1 and DrD2 correlated positively in the striatum (r = 0.908, p < 0.001). Equally, gene expression of CNR1 in the hippocampus correlates positively with OPRM1 (r = 0.500, p < 0.004). However, in the striatum, gene expression of CNR1 correlated positively with DrD2 (r = 0.588, p < 0.001).

a)

## Discussion

In the present work, we explored the effects of the lipid OEA on the reinforcing properties of cocaine using the CPP paradigm. We further supplemented this work using qRT-PCR for characterizing gene expression of four relevant receptors for cocaine reward in the striatum and hippocampus. Our results showed that OEA treatment (10 mg/kg) blocked cocaine-primed reinstatement. In addition, we observed that OEA altered dopaminergic and cannabinoid gene expression. More specifically, decreases in DrD1, DrD2 and CNR1 gene expression levels were detected in the striata of OEA-treated mice compared to those of the CTRL group. In addition, we found a significant decrease in DrD1 gene expression in the hippocampus, but no alterations in other receptors.

Previous studies have observed an attenuating effect of OEA on cocaine reward. In our study, we employed three different administration schedules that differed in timing and number of OEA doses (OEA-C, OEA-EXT and OEA-REINST). Regarding the CPP, we observed that all groups (CTRL, OEA-C, OEA-EXT and OEA-REINST) displayed CPP induced by cocaine (10 mg/kg). A previous report by Bilbao and coworkers [31] showed that coadministration of OEA with cocaine during conditioning reduced the CPP acquisition at 1 and 5 mg/kg doses. In their study, the highest dose of OEA tested (20 mg/kg) completely abolished cocaine-induced CPP (20 mg/kg). To account for these uneven results, it should be noted that, a higher dose of both OEA and cocaine was used than the one employed in our study.

To our best knowledge, this is the first study exploring the effect of OEA administration on cocaine reinstatement. For this purpose, after a series of extinction sessions, mice underwent a reinstatement test induced by a priming cocaine injection (5 mg/kg i.p). We observed that different OEA administration schedules blocked cocaine-primed reinstatement. Interestingly, this effect did not depend on the number of doses received in each experimental group. Mice in the OEA-C and OEA-EXT groups received 4 and 5 doses of OEA respectively, and the OEA-REINST group received only a single dose. However, it is important to remark that in the case of the OEA-REINST group, OEA administration occurred 10 min before the cocaine priming dose. It is possible that administering OEA prior to the cocaine priming dose results in an equivalent effect achieved with a greater number of non-contingent doses (several weeks and 48 h, respectively). Moreover, we must highlight that the OEA-C and OEA-REINST groups required a lower number of sessions to extinguish the preference than the CTRL group (although only OEA-C reached statistical significance). This shorter extinction process suggests that OEA could affect the association of cocaine with a distinctive environment during the conditioning phase in the OEA-C group or accelerate the generation of new learning during extinction in the OEA-EXT group.

Overall, these results are in agreement with several reports of an attenuating effect of OEA on drug-seeking behavior of other substances. Research conducted on primates and rodents have shown that administration of OEA results in decreased nicotine self-administration and reinstatement [42]. Similarly, a previous study used a pharmacological inhibitor of FAAH, the enzyme that catalyzes the hydrolysis of ethanolamides such as anandamide and OEA, to study its effect on cocaine self-administration [43]. Consistent with our results, they found that inhibition of FAAH did not alter cocaine self-administration, but was able to reduce cocaine-seeking behavior on cue-induced and drug-induced reinstatement tests. Interestingly, it has been recently observed that cocaine-induced relapse results in a potent increase in NAEs levels in the striatum but, parallelly, a decrease in tissue levels of OEA in the nucleus accumbens (NAc), cerebellum and hippocampus [34].

Given the mounting evidence of neuroprotective effects of OEA on drug-induced brain damage, we characterized its interaction with gene expression of four receptors of relevant systems mediating cocaine reward and reinstatement.

Dopamine transmission in the striatum is crucial for the reinforcing properties of cocaine [7]. Repeated cocaine administration produces multiple molecular and cellular adaptations, including altered expression of dopaminergic receptor genes in the striatum. Both D1 and D2 receptors mediate the reinstatement of cocaine-seeking behavior. D2 receptor activity generally facilitates priming-induced reinstatement [44], while the use of D2 antagonists diminishes cocaine-primed reinstatement [45]. In the case of D1 receptors, both agonists and antagonists are able to reduce cocaine priming effects [46–48]. Given its role in cocaine reward and reinstatement, we hypothesized that OEA may alter dopaminergic signaling. Indeed, our findings showed reduced expression levels of D1 and D2 in the striatum in OEA-treated mice (OEA-C, OEA-EXT and OEA-REINST) compared to the CTRL group. Similarly, examination of hippocampal gene expression revealed that OEA induced a decrease in D1 receptor in OEA-treated mice. These results are consistent with the reported ability of OEA to restore dopaminergic transmission in the striatum. For instance, Tellez et al., [49] proved that OEA infusion restored the dopaminergic response to fat in mice fed chronically with a high-fat diet. This body of research argues for a role of OEA in regulating basal dopaminergic transmission in the striatum of mice.

Functional studies have demonstrated that TRPV1 activation increases dopaminergic neurotransmission in a variety of brain areas, including the striatum [50]. A recent report has found that administration of a TRPV1 antagonist blocked cocaine CPP reinstatement and decreased D1-like receptor in the NAc, whilst an agonist potentiated cocaine-primed reinstatement [51]. Although the molecular mechanisms have not been identified yet, our results suggest that OEA interacts with dopaminergic signaling in key brain regions for reward processing. Correlation analyses further confirms the important role of D1 receptor in reinstatement of the cocaine place preference as higher hippocampal DrD1 gene expression positively correlated with higher drug-induced reinstatement scores.

We also observed reduced expression of the CNR1 receptor in OEA-treated mice compared with the CTRL group. The major receptors responsible for cannabinoid-mediated effects are CB1 and CB2. A large body of research confirms the involvement of endocannabinoid lipids in the modulation of dopaminergic transmission and cocaine-induced reinstatement. CB1 receptors are present in high density in the striatum [52, 53]. It has been observed that the cannabinoid system modulates dopaminergic signaling mainly by acting on the TRPV1 receptor [54, 55]. As a non-cannabinoid NAEs, OEA does not directly bind to CB1 or CB2 but can potentiate the effects of other lipid messengers such as anandamide (“entourage effect”) [56]. The reduction in gene expression of CNR1 observed in our study suggests a pharmacological effect of OEA on cocaine-priming. We hypothesize that OEA treatment alters the activity of CB1 receptors and ultimately reduces dopaminergic signaling in response to a single priming dose of cocaine. The correlation analyses also support this hypothesis. Results show a tendency towards a positive correlation between CNR1 gene expression and drug-induced reinstatement scores so that lower CNR1 gene expression in the hippocampus was correlated with a lower reinstatement preference. Of note, we did not observe any changes in the µ opioid receptor gene expression. To our knowledge, there are no reports of an OEA effect on the opioid system.

Our present understanding of OEA bioactivity includes binding to PPARα receptor and TRPV1. It is possible that OEA attenuates drug-induced reinstatement through D1 and D2 receptor activity in the striatum and hippocampus since there is a high density of PPARα receptors in these brain regions [57]. It is important to remark that dopaminergic receptors are constantly adapting to the changing extracellular dopamine concentrations and thus, changes in receptor mRNA expression reflect their specific activity at the time of tissue collection for analysis. In addition, it is important to consider that mRNA expression does not necessarily correlate with the activity, affinity or sensitivity of the different receptors. Considering these shortcomings, further research should explore the specific pathways where OEA acts to counteract drug-induced dopamine plastic changes contributing to a greater risk of reinstatement.

In conclusion, our findings further support the previous pharmacological evidence of a modulatory effect of OEA on reward behavior. The present study shows that different OEA treatments prevent cocaine reinstatement mainly by modulating overall dopaminergic signaling in limbic areas including the striatum and hippocampus. Although the neuronal mechanisms have not been clearly defined, our results offer strong evidence of an attenuating effect of OEA on cocaine reinstatement.

## Data Availability

The data are available for any scientific use from the corresponding author on reasonable request.
